# Colon bypass with a colon-flap augmentation pharyngoesophagoplasty

**DOI:** 10.11604/pamj.2015.21.275.6717

**Published:** 2015-08-11

**Authors:** Mark Tettey, Frank Edwin, Ernest Aniteye, Martin Tamatey, Ekow Entsua-Mensah, Ernest Offosu-Appiah, Innocent Adzamli

**Affiliations:** 1 National Cardiothoracic Centre, Korle Bu Teaching Hospital, Accra, Ghana; 2 University of Ghana School of Medicine and Dentistry, Ghana

**Keywords:** Pharyngo-esophageal stricture, colon-flap, pharyngo-esophagoplasty, dysphagia

## Abstract

Extensive caustic stricture of the upper aero-digestive system (oro- and hypo-pharynx) is a severe injury with limited surgical options. We adopted augmentation of the cicatrized upper aero-digestive tract with colon as our preferred management option. The aim of this report is to describe our initial experience with the technique of colon-flap augmentation pharyngo-esophagoplasty (CFAP) for selected patients with severe pharyngo-esophageal stricture. Between October 2011 and June 2013, three male patients (aged 16, 4 and 18 years respectively) underwent CFAP following extensive pharyngo-esophageal stricture. Postoperative recovery was uneventful in all three cases and all started swallowing within 7 - 10 days after surgery without significant dysphagia. Colon-flap augmentation pharyngo-esophagoplasty is an effective procedure for reconstruction of the pharynx and the hypopharynx after extensive caustic pharyngoesophageal structure in selected cases.

## Introduction

In modern medicine, innovation is paramount in the pursuit of optimal care and best practice in managing patients with difficult and rare abnormalities. The management of severe pharyngoesophageal strictures brings to the fore the challenge of doing everything possible for patients to regain normal or near normal swallowing. Extensive pharyngo-esophageal tissue necrosis and subsequent scarring permanently destroys the normal swallowing mechanism. Invariably associated with severe pharyngeal stricture is complete stricture of the esophagus. Pharyngoesophagoplasty interferes with normal mechanisms for airway protection setting the stage for aspiration during swallowing. After pharyngoplasty, rehabilitative training to allow deglutition without aspiration is imperative; near normal swallowing may take between 5-12 months of rehabilitative training [1, 2]. Pharyngeal involvement in caustic strictures accounts for 4.4% of the caustic strictures treated at the National Cardiothoracic Center in Accra [1]. Different procedures have been described in the management of this particular group of patients with varying results. The use of colon pedicled on the left or the right colic arteries, stomach, ormyocutaneous flaps have been described [2, 3]. We have described suprahyoid colopharyngoplasty for some of these intractable cases of severe pharyngoesophageal strictures [1]. The peculiar presentation of pharyngoesophageal stricture in a sixteen year old boy prompted our currenttechnique. The purpose of this communicationis to share our experience withthe technique of colon-flap augmentation pharyngoesophagoplasty (CFAP).

## Methods

Three patients who presented with severe pharyngeal strictures with hypopharyngealstenoses, intact laryngeal openings and complete strictures of the esophagus form the basis for this report. Their ages were Case A: 16 years, Case B: 4 years and Case C: 18 years. All accidentally ingested caustic soda and presented with absolute dysphagia. Feeding gastrostomy for nutritional rehabilitation was performed in each case after initial resuscitation. Duration from caustic ingestion to the time of surgery was 9 months for the Case A and more than 12 months for Cases B and C.

**Figure 1:**
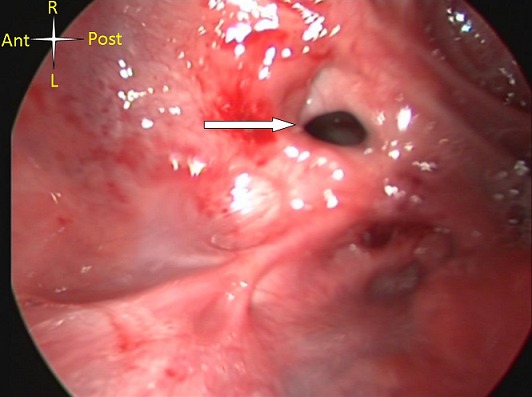
severe caustic injury of the pharynx: note the laryngeal opening (arrow)

**Patient Selection**: all patients sustained severe pharyngoesophagealstricture, hypopharyngeal stenosis with an intact laryngeal opening. Diagnostic contrast imaging was challenging due to swallowing difficulties. The decision to perform CFAP was taken intra-operatively in each case - hypopharyngeal stenosis with an intact larynx was an absolute indication. For more severe injuries resulting in complete hypopharyngeal obstruction, a suprahyoid colopharyngoplastywould beperformed instead as described previously [1]. Severe caustic injury of the upper aerodigestive tract creates several structural defects (Figure 1) scarred destroyed epiglottis, oropharyngeal and laryngotracheal stricture, restricted movement of some of the oropharyngeal muscles caused by extensive scarring, and severe laryngeal stricture; most of these patients end up with permanent tracheostomy. None of the three patients in this reportsustained severe laryngeal stricture.

**Pre-operative Preparation**: nutritional status assessment was carried out. Body mass index-for-age percentile was between 5^th^ and 10^th^ percentile in each of the three cases. The hemoglobin for Cases A, B & C were 12.2g/dl, 11.5g/dl and 11.9g/dl respectively. Renal and liver function tests were normal in all three cases. Bowel preparation before surgery was carried out routinely.

**Anaesthesia:** general anesthesia was used in all three cases. Invasive monitoring was mandatory to ensure stable hemodynamics throughout surgery and prevent fluid overload during surgery. Patients were positioned supine with the neck extended. Adequate exposure of the neck was vital in enhancing dissection.

**Figure 2:**
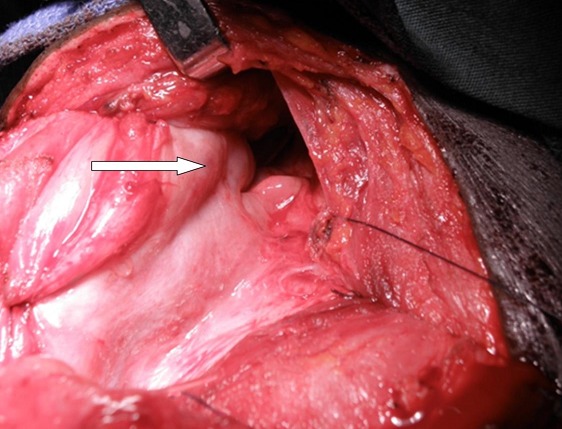
stenosed hypopharynx completely incised: note the interarytenoid notch (arrow)

**Figure 3:**
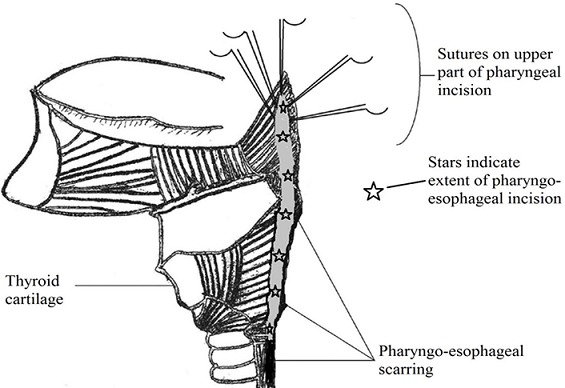
placement of five stay sutures at the proximal end of the pharyngeal incision to help anchor the flap

**Figure 4:**
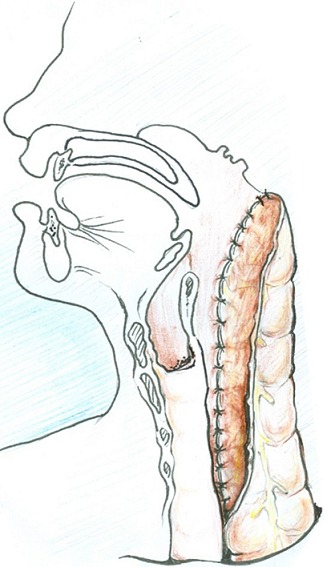
colon-flap augmentation pharyngoesophagoplasty: pharyngeal reconstruction is carried out with the colon-flap after incision in the pharynx, as shown in figure 3

**Surgical Technique:** the neck dissection is first carried out after routine cleaning and draping; a J-shaped skin incision starting from the suprasternal notch and extending medial to the left sternocleidomastoid muscle. This incision is deepened carefully to expose the esophagus. The pharynx is mobilizedfrom the vertebrae proximally up to the angle of the left mandible. The anesthetist then introduces a nasogastric tube which helps to locate the proximal extent of the stricture as the nasogastric tube is moved within the lumen. A longitudinal incision is made above the stricture close to the median raphe in the pharynx. The hypopharynx is completely laid open; this incision is extended into the pharynx at least 2 cm above the level of the laryngeal opening. At this point the endotracheal tube is visible as it enters the larynx (Figure 2). Having performed the dissection thus far, attention shifts to the abdomen. Through a median laparotomy, the left colon and its arterial supply are assessed. The length of colon to be harvested for replacing the esophagus and reconstruction of the pharynx is determined by the distance between the origin of the left colic artery and the proximal extent of the pharyngeal incision. This may extend from the originof the left colic artery to the originof the right colic artery. The harvested colon is pedicled on the left colic artery. Colonic continuity is established by end-to-end colo-colic anastomosis. The distal end of the harvested colon is anastomosed to the body of stomach to create an isoperistaltic anastomosis when the proximal end is subsequently used to reconstruct the pharynx. A retrosternal tunnel is created by blunt dissection from the posterior aspect of the xiphisternumemerging just behind the suprasternal notch. The proximal end of the colon is then routed through this tunnel to the neck. Care is taken not to twist the vascular pedicle in the process. The length of the pharyngeal incision is measured to determine the length of colon flap to be created. A longitudinal incision is made at the antimesenteric border of the colon to correspond with the length of the pharyngeal incision. Five stay sutures are placed at the apex of the pharyngeal incision (Figure 3). This is used to anchor the colon initially before posterior interrupted sutures are placed followed by anterior interrupted sutures (Figure 4). Resection of scar tissue was avoided in all cases. The colon flap created at the proximal end is used to enlarge the oropharynx and the stenosed hypopharyngeal space. The neck and abdominal incisions are closed in two layers. Postoperatively, food swallowed passes through the enhanced hypopharynx into the interposed colon without significant laryngeal contamination oraspiration. The gastrostomy tube is maintained for decompression and feeding post-operatively.

**Post-operative care**: patients were admitted to the ICU for continuous monitoring following extubation in theatre. Hypotension was avoided in the immediate postoperative period by paying attention to volume status. Vessels supplying the harvested colon could go into spasm with hypotension and this can affect the integrity of the anastomosis. Total parenteral nutrition was used in all patients in the early postoperative period before commencement of nasogastric tube and oral feeding on the 10^th^ day.

## Results

Case A had difficulty swallowing liquids (mild aspiration with cough) on the tenth day. A barium swallow study showed inadequate oropharyngeal flap augmentation with stenosis at the level of the larynx. This was related to the shorter extent of the pharyngeal incision (an important learning point which was successfully applied in cases B&C). However, case A started swallowing without significant aspiration 14 days after discharge. Cases B and C started swallowing without aspiration on the 10^th^ post-operative day when feeding was commenced. Post-operative recovery was uneventful in all three cases with no complications. Case A was discharged on postoperative day 14 while cases B&C were discharged on the 12^th^ post-operative day.

**Follow-up**: Case A: 2 years; Case B: 18 months; Case C: 2 months. As at the time of writing this report, all three patients tolerated normal diet with no significant dysphagia.

## Discussion

The severity of stricture caused by caustic ingestion depends on a number of factors - the type of substance ingested the concentration of the substance, the volume, and the contact time [4]. Alkali ingestion causes liquefactive necrosis with diffusion into deeper layers of the injured mucosa. This injury occurs quickly with a 30% solution of sodium hydroxide being able to produce full thickness injury in 1 second [4]. In developing countries where the sale of caustic soda for domesticsoap making is unregulated, this substance in the liquid form is ingested accidentally in various concentrations. Up to about 4.4% of patients presenting toour center have varying degrees of pharyngeal strictures with concomitant esophageal strictures [1]. The three patients reported developed some degree of oropharyngeal stricture, hypopharyngeal stenosis and complete stricture of the esophagus. The laryngeal opening was intact in all cases and all had endotracheal intubation without difficulty. The challenges faced in this category of patients include: reconstruction of the scarred pharynx without compromising upper airway protective mechanisms, coordinated swallowing to simulate normal deglutitionand restoring a near normal pharyngeal space after postoperatively. Colon interposition usingCFAP helped to volume enhance the pharynx and the hypopharynx and maintained coordinated swallowing since part of the stenosed hypopharynx anatomically was kept intact in the reconstruction of the hypopharynx. The flap created enough space in the pharynx and hypopharynx helping to prevent aspiration soon after the patients started swallowing on the tenth day. Scar resection was notperformed. Scar resection was an integral part of the colopharyngoplasty described by Chirica et al, [2]. Resection of scar tissue in our category of patients will mean excising the stenosed hypopharynx and performing a suprahyoid colopharyngoplasty. In our experience, the postoperative recovery following suprahyoid colopharyngoplasty is more protracted [1]. Even though the colon was sutured to the scarred hypopharynx and pharynx, there was no fistula formation ten days after the patients started swallowing. The technique of colopharyngoplasty used in CFAP is similar to what was first described by Popovici as “pharyngoplastiavera” [5]. Their procedure was based on the reconstruction of the 3 walls of the hypopharynx by lining it with visceral material after complete separation of the larynx from the pharynx. The difference is that reconstruction in CFAP was performedin patients with a functioning larynx; the colon-flap created was used to enhance the contracted and stenosed hypopharynx and the pharyngoplasty was carried out by a colon-flap sutured to the scarred tissues followinga longitudinal incision in the hypopharynx and the pharynx. A good functional result was evident upon initiation of swallowing (or following a two weeks delay in Case A). The cause of the delayed functional recovery in Case A was judged to be the relatively shorter oropharyngeal incision. Fluoroscopic imaging showed that in the course of the swallowing reflex, the laryngopharynx narrowed as the larynx was elevatedso that contrastspilledanteriorly into the larynx. With this insight, the pharyngeal incision was extended in the subsequent two cases with much better functional results. Currently, all three patients are tolerating normal diets and are completely satisfied with the outcome.

## Conclusion

The functional result of colon-flap augmentation pharyngoesophagoplasty is very good with excellent medium term results. The procedure is simple to perform and could be used in patients with extensive pharyngeal stricture, stenosis of the hypopharynx andan intact laryngeal opening.

## References

[1] Mark Tettey, Frank Edwin, Ernest Aniteye, Martin Tamatey (2011). Colopharyngoplasty for intractable caustic pharyngoesophageal strictures in an indigenous african community? adverse impact of concomitant traceostomy on outcome. Interact Cardivasc Thorac Surg.

[2] Mircea Chirica, Cecile de Chaisemartin, Nicolas Goasguen, Nicolas Munoz-Bongrand (2007). Colopharyngoplasty for the treatment of severe pharyngoesophageal caustic injuries: an audit of 58 patients. Ann Surg.

[3] Ti TK (1980). Esophageal resection with pharyngogastrostomy for corrosive stricture of the pharynx and the esophagus. Br J Surg.

[4] Michael Lupa, Jaqueline Magne J, Lindhe Gaurisco, Ronald Amedee (2009). Update on diagnosis and treatment of caustic ingestion. Oschhsner J.

[5] Zeno Popovici (1989). About reconstruction of the pharynx with colon in extensive corrosive stricture. The Kurume Med J.

